# DNA barcoding of euryglossine bees and the description of new species of *Euhesma* Michener (Hymenoptera, Colletidae, Euryglossinae)

**DOI:** 10.3897/zookeys.520.6185

**Published:** 2015-09-16

**Authors:** Katja Hogendoorn, Mark Stevens, Remko Leijs

**Affiliations:** 1 School of Agriculture, Food and Wine, The University of Adelaide, SA 5005; 2 South Australian Museum, North Terrace, Adelaide, SA 5000; 3 School of Pharmacy and Medical Sciences, University of South Australia, Adelaide, SA 5000

**Keywords:** barcoding, species discovery, euryglossine bees, Bush Blitz survey, conservation

## Abstract

This paper launches an open access DNA barcoding project “AUSBS” under the Barcoding of Life Datasystems (BOLD). The aims of the project are to help scientists who lack the necessary morphological knowledge to identify known species using molecular markers, to aid native bee specialists with the recognition of species groups that morphologically are difficult to define, and, eventually, to assist with the recognition of new species among known species. Using integrative taxonomy, i.e. morphological comparison to type specimens in Australian museum collections combined with phylogenetic analysis of a fragment of the mitochondrial DNA cytochrome *c* oxidase subunit I (mtCOI) gene sequences led to the recognition of four new species of *Euhesma* Michener (Hymenoptera: Colletidae: Euryglossini) collected during intensive surveys in remote Australian conservation areas, which are described. The new species are *Euhesma micans*, *Euhesma lyngouriae*, and *Euhesma aulaca* in a species group associated with *Eremophila* flowers, and *Euhesma albamala* in the *walkeriana* species group.

Barcoding of Life Datasystems

mitochondrial DNA cytochrome *c* oxidase subunit I

## Introduction

Australia is facing a dramatic and unprecedented loss of biodiversity (Ritchie et al. 2013). However the main substance for this contention is derived from data on native vegetation (Bradshaw 2012) and vertebrates (Department of the Environment 2009). Despite the numerical dominance of invertebrates, their fundamental importance in natural ecosystems and their services to agricultural production (Tscharntke et al. 2012), changes in their biodiversity are not well documented in Australia. This lack of information about the conservation status of Australian invertebrates is at least partly caused by a lack of knowledge of taxonomy, distribution and population dynamics of Australian invertebrate fauna (New and Sands 2004). What is not known cannot be monitored.

Australian native bees serve as a case in point. Despite their environmental and economic importance as pollinators of native plants (e.g. Houston et al. 1993), and as ecosystem services providers for crop pollination worldwide (Garibaldi et al. 2013) and in Australia (Keogh et al. 2010), only an estimated two-thirds of the Australian bee species are as yet known to science (Batley and Hogendoorn 2009). Opportunities to ameliorate this situation are constrained by a shortage of funding and career prospects for taxonomists. Hence, species may become extinct before they have been recognized.

To make native bees more accessible to the scientific community, an open access project “AUSBS” has been initiated in the Barcoding of Life Datasystems (BOLD, Ratnasingham and Hebert 2007). The aim of the project is twofold. Firstly, it will allow scientists with molecular capability but insufficient knowledge of bee taxonomy and systematics to recognize species and document local biodiversity of native bees. Secondly, for Australian native bee taxonomists, it contributes to integrative taxonomic approaches, such as elucidation of related species and clarification of problematic species groups, association of the sexes within one species, the association of larvae with adults and the identification of new species (Gibbs 2009; 2011; Packer et al. 2009; Schmidt et al. 2015).

Naturally, this initiative will only be successful if there is a wide coverage of species in the barcoding database. It is intended to regularly update the database with entries of additional species. So far, 271 sequences have been added to the project, covering 120 species in four of the five Australian bee families (Megachilidae, Apidae, Colletidae and Halictidae) that were collected during Bush Blitz surveys – intensive short-term surveys of remote, protected areas funded by the Australian Federal Government (Department of the Environment 2010).

In this publication the results of DNA barcoding are focused on species within the Euryglossinae (Colletidae). This subfamily has been relatively well studied through descriptions of new species by Houston (1992) and numerous revisions of genera by Exley (1998; 2001; 2002; 2004) and keys to the species are available for several genera and species groups. The DNA sequence data submitted to BOLD was used to assist in delineating species into species-groups in the genus *Euhesma* (Michener), (1965) and to identify the sexes that comprise a species. Subsequent morphological comparison with type specimens allowed the recognition of four new species, which are described. The species selected for description belong to two well-delineated species groups within *Euhesma*: the *walkeriana* species group (Exley 2001), and the group associated with *Eremophila* (Myoporaceae; Exley 1998). Brief morphological characteristics of these groups are given below.

## Methods

The bee specimens studied in this paper were collected during six intensive short term (1–2 week) Bush Blitz surveys (Department of the Environment 2010), at various remote locations in Australia (AUSBS 2014). Coordinates of the locations are given in decimal degrees (Suppl. material 1, Table 1). The species were caught mainly on flowering plants using a hand net, but on occasion a vehicle net or malaise traps were used. The bees were killed by freezing, pinned within a day of capture and sorted into morpho-species.

For DNA analyses a single middle leg was removed from up to five specimens per morpho-species. These legs were stored in 100% ethanol to allow preservation of the DNA, and submitted to BOLD for DNA barcoding using the cytochrome *c* oxidase subunit 1 gene. Specimen details, including collecting dates and locality information can be accessed in BOLD under the project Australian Bee Survey (accession numbers AUSBS001-12 to 190-12 and AUSBS191-13 to 380-13, e.g. http://www.boldsystems.org/index.php/Public_RecordView?processid=AUSBS131-12, and http://www.boldsystems.org/index.php/Public_RecordView?processid=AUSBS205-13). Using only BOLD BIN compliant sequence data (Ratnasingham and Hebert 2007) as input, species were delineated using Neighbor-joining trees generated by BOLD and uncorrected sequence divergence data were calculated using PAUP* version 4.0b8 (Swofford 2001). A phylogenetic tree of the barcoded data was generated using MrBayes version 3.2 (Ronquist et al. 2012).

We attempted to key all collected euryglossine specimens to species. The specimens were compared to all type specimens and all other relevant reliably named material at the Queensland Museum, the Western Australian Museum, the Australian National Insect Collection, and the South Australian Museum. Based on these morphological comparisons, several species, including four species in the genus *Euhesma*, were identified as new. These four species are described here.

The descriptions of the species and the morphological terminology follow the format used by Exley (2001) for the *walkeriana* species group and Exley (1998) for the species associated with *Eremophila* (Myoporaceae), to allow easy comparison with the other species in the same species groups. Stereomicroscope with step-less zoom and an eyepiece micrometer were used to take relative measurements of the head (following Houston 1990), whereby head width was set to 50 units.

### Abbreviations

BOLD Barcoding of Life Database

SAMA South Australian Museum, Adelaide

WAM Western Australian Museum

## Results

### Molecular delineation of the taxa

**Figure 1.**
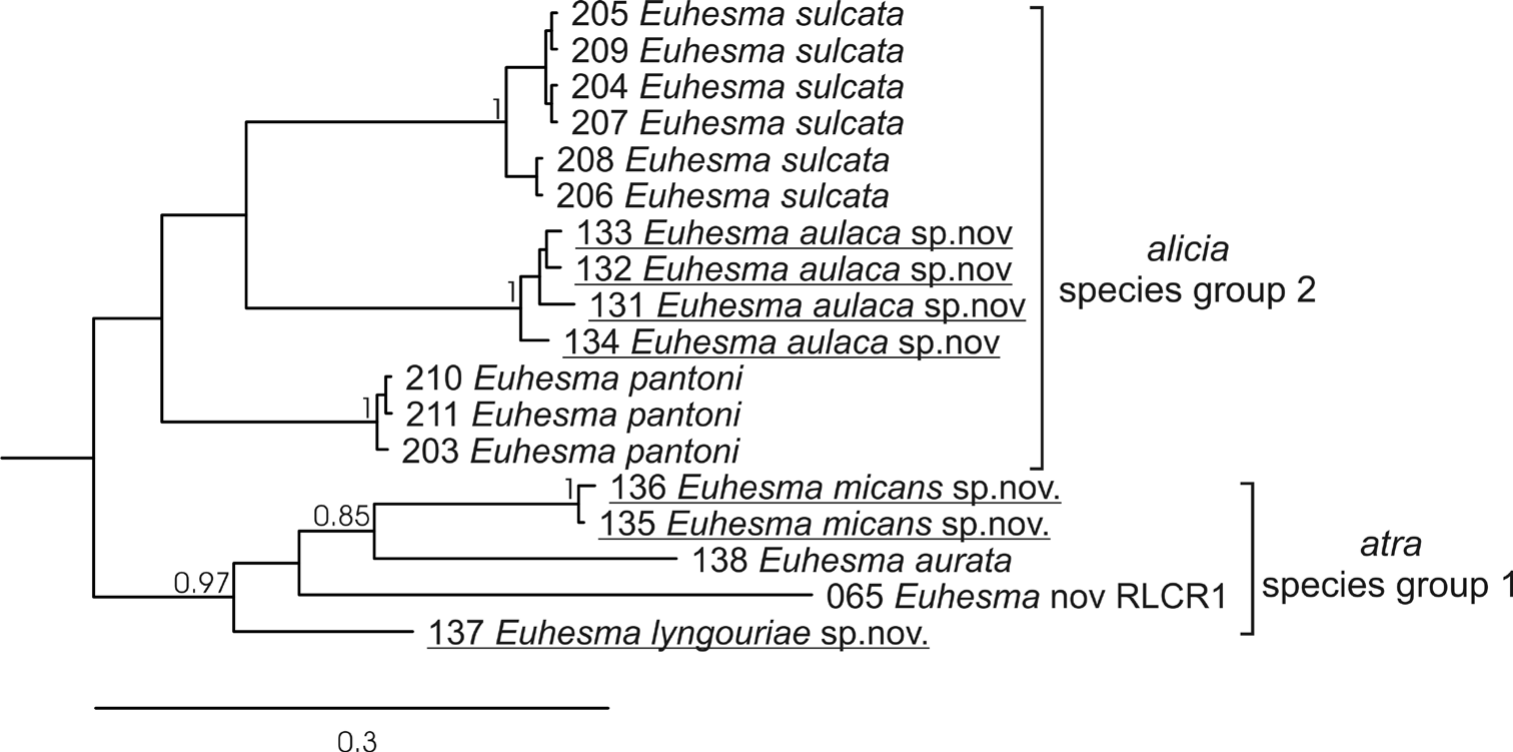
Phylogenetic relationships among *Euhesma* species collected on *Eremophila* flowers, based on BOLD sequence data, analysed using MrBayes (GTR-inv+gamma, partitioned by codon, 8M generations). Posterior probabilities for nodes are shown when > 0.7. The species described in this publication are underlined. The three digits preceding taxon names refer to Barcoding of Life Database: AUSBS###-12/13 specimens (AUSBS 2014).

BOLD barcoding of Euryglossinae resulted in DNA barcode data for 87 specimens comprising 40 species. Of these, morphological examination resulted in the identification of 17 species, 6 species were recognized as new and 17 species were identified to genus or species group level (Suppl. material 2, Fig. 1). The molecular delineation matched the morphologically identified genera and species groups for the species in the genera *Euryglossa*, *Euryglossula*, *Euryglossina*, *Pachyprosopis*, *Hyphesma*, *Xanthesma*, *Pachyprosopis*, and some species groups within *Euhesma*. However, *Euhesma* and *Callohesma* appear to be paraphyletic (Suppl. material 2, Fig. 1).

### New species

A number of specimens were identified to belong to *Euhesma* species groups that previously were revised by Exley (1998, 2001). Morphological comparison of these specimens with species descriptions and with type material in the above mentioned Australian museum collections showed that, among a number of known species, five of the *Euhesma* species did not match with any of the descriptions nor with any of the examined type specimens. Molecular and morphological data showed that four of these species belong to existing species groups. These four species are described in the systematics section of this paper.

One of the four new species described here belongs to the *Euhesma walkeriana*-species group (Exley 2001, Suppl. material 2, Fig. 1). The pairwise uncorrected sequence divergence between the new species (*Euhesma albamala* sp. n.) and the only other barcoded species (*Euhesma bronzus* Exley 2001) in this group varied between 5.0–6.9%.

Three other new species belong to a group of *Euhesma* species that are associated with flowers of *Eremophila* (Exley 1998). Pairwise uncorrected sequence divergence between phylogenetic sister species in this group are: *Euhesma sulcata* Exley 1998 vs. *Euhesma aulaca* sp. n. 5.0-6.1%; *Euhesma lyngouriae* sp. n. vs. *Euhesma micans* sp. n. 5.8–6.1%; and, *Euhesma aurata* Exley 1998 vs. *Euhesma micans* sp. n. 6.0–6.3%. Because the data sets for the above species groups are incomplete, the presented divergence values are not necessarily comparisons with phylogenetically closest sister species.

While the molecular results group a fifth species of *Euhesma*, represented by a single barcoded male (RL1788A-AUSBS065) and some additional male and female specimens, with *Euhesma* species caught on *Eremophila*, it has a number of characters that are not consistent with this group, i.e. head and mouth parts not elongated and not caught on *Eremophila*. Thus, this species did not fit into any of the other known *Euhesma* groups and therefore the description was deferred until additional data allow improved justification for the position of this species relative to other *Euhesma* species.

## Systematics

### Four new species of *Euhesma* Michener

Within the subfamily Euryglossinae, the genus *Euhesma* Michener contains a large number (65) of highly diverse species (Michener 2007). The genus was erected by Michener (2000) as a “dumping ground” (Exley 2001) for species that do not fit easily elsewhere. As a result, the genus is difficult to delineate (Exley 2001, 2004). To provide more structure and delineation within the genus, Exley (1998, 2001, 2004) divided the genus into a series of species groups, which so far account for approximately half of the species included in the genus. These groups include the *walkeriana* species group (15 species; Exley 2001), the *acantha* species group (three species; Exley 2004), and three groups associated with the plant *Eremophila* (Exley 1998): the *atra*, *alicia* and *coppinensis* species groups (20 species combined).

### A new species of *Euhesma* in the *walkeriana* species group

The fifteen known species in the *walkeriana* species group (Exley 2001) are small (4–6 mm), black and or with metallic sheen, often marked with yellow. Heads are wider than long with antennae low down on the face so that subantennal sutures are absent or almost so. While similar to Xanthesma (subgenus Chaetohesma), the *walkeriana* group differs from this subgenus in the following characters: the facial foveae are straight and do not curve towards lateral ocelli, the pronotum is relatively short and the basitarsi of the forelegs do not bear long, stiff setae (Exley 2001).

As only a single species is added to this group, we do not produce a modified key, but suggest modifying the key produced by Exley (2001) by inserting additional couplets after couplet 3 as follows:

**Table:** 

4 (3)	Labrum yellow	**5**
–	Labrum brown/black	**5A**
5A (4)	Mandibles transparent white with dark tips	***Euhesma albamala* sp. n.**
–	Mandibles yellow, brown or black	**6**

#### Euhesma albamala

Taxon classificationAnimaliaHymenopteraColletidae

Hogendoorn & Leijs sp. n.

http://zoobank.org/C018DBB3-3224-40F5-A1AD-F45D7E79A083

Figs 2A–H , 3A–D

##### Material examined.

*Holotype:* ♀, RL1807C, Cane River Conservation Park, Western Australia, 22.0936°S, 115.3507°E, 26 June 2011, R. Leijs, on flowers of a red flowering *Grevillea* (WAM). BOLD: AUSBS082-12. *Paratypes:* ♂, RL1811, same date and locality as holotype (WAM). BOLD: AUSBS009-12; 4♀, all same date and locality as holotype (SAMA 32-033287, -288, -289, -290).

##### Diagnosis.

Most like *Euhesma spinola*; however, the combination of the white mandibles with dark tips and the simple claws distinguishes this from all other species. The absence of both pothook and clubbed setae is a character shared with *Euhesma lobata*, *Euhesma spinola* and *Euhesma walkeriana*.

##### Description.

*Female.* Length approximately 5.0 mm; wing length about 3.2 mm. Head width 1.5 mm. Relative head measurements: width 50, length 41; clypeal length 11; lower interocular distance 30; upper interocular distance 28; interantennal distance 8; antennocular distance 7; interocellar distance 10; ocellocular distance 8.

Supraclypeal area, frons and mesosoma with metallic bronze-green sheen; mandibles translucent white with dark tips; labrum brown; legs amber with coxae and tibiae dark brown, and hind basitarsi pale yellow; face with long white hairs; forelegs with coxal lobes weakly developed with long hyaline unbranched setae; clubbed and pothook setae absent; hind basitibial plate with carina; hind-tibia with a row of tubercles beyond basitibial area; claws simple; metasoma brown with posterior margins of terga translucent, creating a banded effect; pygidial plate broadly spathulate.

*Male.* Length approximately 3.8 mm; wing length approximately 3 mm. Head width 1.2 mm. Relative head measurements: width 50, length 45; clypeal length 10; lower interocular distance 30; upper interocular distance 30; interantennal distance 8; antennocular distance 7; interocellar distance 10; ocellocular distance 8. Coloration and pubescence as in female, but pronotum with metallic purple sheen; metasoma dark brown with posterior margins of terga translucent. The apical five flagellar segments of the male look crumpled, however this could be an artifact. Terminalia as in Fig. 3A–D.

**Figure 2.**
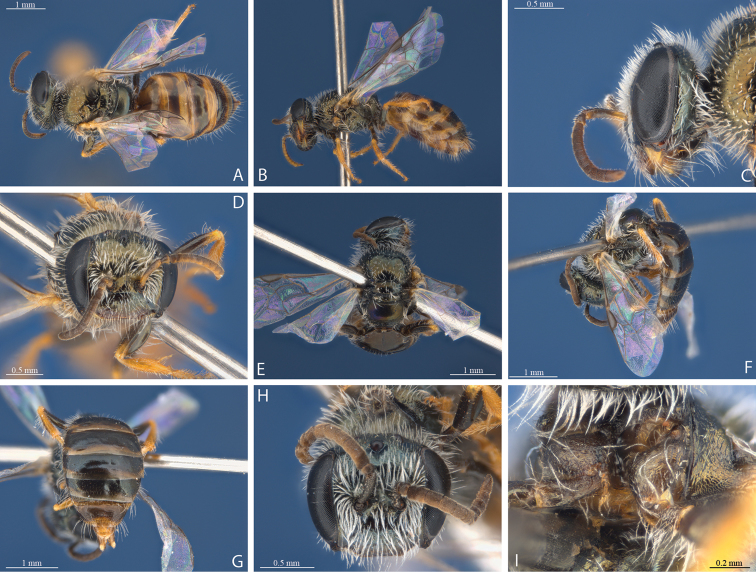
*Euhesma albamala* sp. n.: **A–D** Holotype female: **A** dorsal **B** lateral **C** head lateral **D** head frontal **E–H** Allotype male: **E** dorsal **F** lateral **G** tergites **H** head frontal. Fore coxae female.

**Figure 3.**
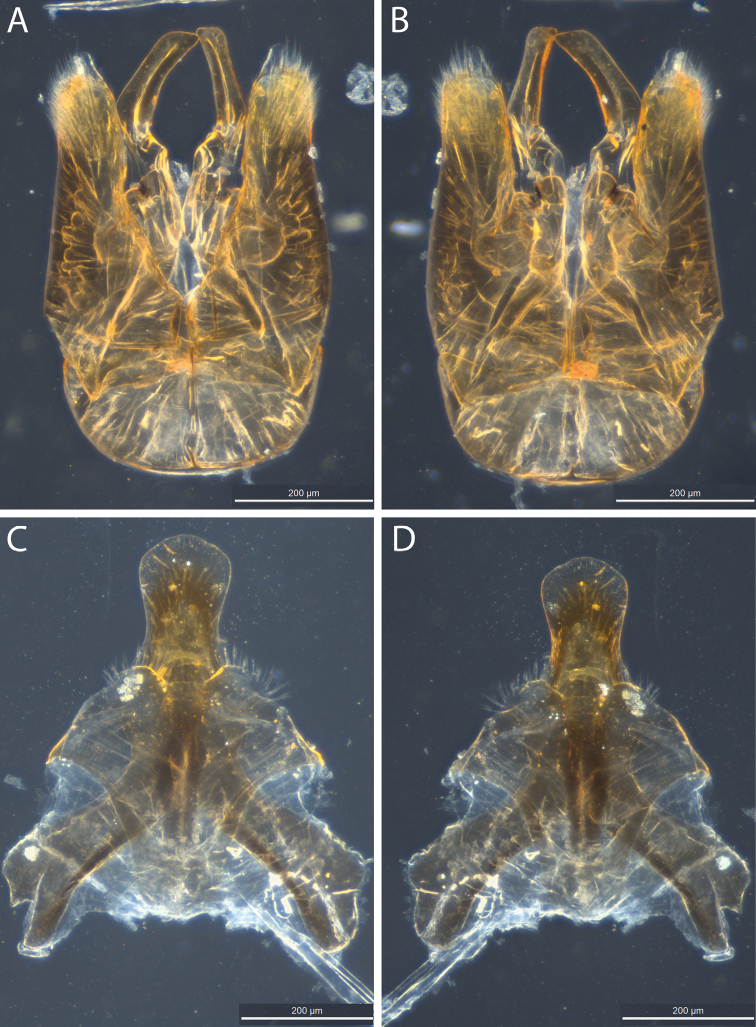
*Euhesma albamala* sp. n.: **A–D** Allotype male genital structures: **A** genital ventral **B** genital dorsal **C** S7+S8 ventral **D** S7+S8 dorsal. Scale bars: 200 µm.

##### Etymology.

The specific name refers to the white mandibles.

### Three new species of *Euhesma* associated with *Eremophila*

The bees in the three known species groups associated with *Eremophila*, the *atra* (eight species), the *alicia* (eight species) and the *coppinensis* (four species) species groups, have modified heads and/or labial palps that suggest foraging on narrow, tubular flowers (Exley 1998). The distinction between the species groups is based on the total length of the labial palps, and on the relative length of segment 2 and 4 of these palps (Exley 1998). Of the three new species with similar adaptations described here, two species, i.e. *Euhesma micans* and *Euhesma lyngouriae* belong in the *atra* species group. The remaining new species, *Euhesma aulaca*, belongs in the *alicia* species group.

To allow easy identification of the *Euhesma* species associated with *Eremophila*, Exley’s (1998) key to the species of *Euhesma* collected on *Eremophila* is modified to incorporate the three new species.

### Key to the species of *Euhesma* collected on *Eremophila*

**Table:** 

1	Labial palps enormously extended, nearly as long as or longer than head	**2**
–	Labial palps much shorter than head	***atra*-group 14**
2(1)	Labial palps with segment 2 much shorter than segment 4	***alicia*-group 6**
–	Labial palps with segment 2 subequal to or longer than segment 4	***coppinensis*-group 3**
3(2)	Supraclypeal area and clypeus medianly concave; body length about 5 mm	***Euhesma walkeri***
–	Neither supraclypeal area nor clypeus medianly concave; body length about 6 mm	**4**
4(3)	Labial palps with segments 2 and 4 subequal in length	**5**
–	Labial palps with segment 2 clearly longer than segment 4	***Euhesma***
5(4)	Labial palps longer than head	***Euhesma macrayae***
–	Labial palps as long as head	***Euhesma newmanensis***
6(2)	Labial palp segment 3 clearly longer than segment 4	**7**
–	Labial palp segment 3 not clearly longer than segment 4	**8**
7(6)	Supraclypeal area, clypeus medianly and frons medianly concave	***Euhesma pantoni***
–	Supraclypeal area, clypeus and frons not concave	***Euhesma alicia***
8(6)	Labial palp segments 3 and 4 together much shorter than head	**9**
–	Labial palp segments 3 and 4 together about as long as head	**11**
9(8)	Upper margin of clypeus indistinct	***Euhesma aulaca* sp. n.**
–	Upper margin of clypeus distinct	**10**
10(9)	Body length about 5 mm	***Euhesma yellowdinensis***
–	Body length about 6 mm	***Euhesma wiluna***
11(8)	Tibiae of all legs golden	***Euhesma cuneifolia***
–	Tibiae of all legs mostly dark brown	**12**
12(11)	Fronto-clypeal suture clearly evident	***Euhesma meeka***
–	Fronto-clypeal suture not clearly evident	**13**
13(12)	Clypeus with a median longitudinal furrow	***Euhesma sulcata***
–	Clypeus without a median longitudinal furrow	***Euhesma granitica***
14(1)	Only known from Northern Territory and Queensland	***Euhesma sturtiensis***
–	Only known from Western Australia and South Australia	**15**
15(14)	Tibiae, tarsi and terminal gastral terga golden brown	**16**
–	Tibiae, tarsi and terminal gastral terga predominantly dark brown	**17**
16(15)	First recurrent vein distal to first submarginal crossvein	***Euhesma aurata***
–	First recurrent vein interstitial with first submarginal crossvein	***Euhesma lyngouriae* sp. n.**
17(15)	Labial palp longer than antennal flagellum	**18**
–	Labial palp not longer than antennal flagellum	**19**
18(17)	Supraclypeal area almost glabrous and highly polished	***Euhesma symmetra***
–	Supraclypeal area neither glabrous nor polished	***Euhesma nalbarra***
19(17)	Dorsal surface of clypeus concave medianly (indented)	***Euhesma atra***
–	Dorsal surface of clypeus with a slight median longitudinal furrow	***Eremophila scoparia***
–	Dorsal surface of clypeus in no way concave	**20**
20(19)	Labial palps clearly longer than maxillary palps	***Euhesma leonora***
–	Labial palps and maxillary palps about equal in length	**21**
21(20)	Fronto-clypeal suture distinct	***Euhesma balladonia***
–	Fronto-clypeal suture absent on clypeus	***Euhesma micans* sp. n.**

#### Euhesma micans

Taxon classificationAnimaliaHymenopteraColletidae

Hogendoorn & Leijs sp. n.

http://zoobank.org/CD2BD7B3-F09C-4DD4-B953-EE4326804955

Figs 4A–I , 5A–E

##### Material examined.

*Holotype:* ♀, SAMA 32-03385, Bon Bon Station, South Australia, 30.5250°S, 135.5917°E, 27 October 2010, R. Leijs, on flowers of *Acacia victoriae* (SAMA). BOLD: AUSBS135-12. *Paratype:* ♂, SAMA 32-03386, same date and locality as holotype (SAMA). BOLD: AUSBS136-12.

##### Diagnosis.

Most like *Euhesma leonora*; however this is the only species in which the frons above the antennae is shining, and the facial fovae are narrow and not curved towards the ocelli.

##### Description.

*Female.* Length approximately 4.5 mm; wing length approximately 3.1 mm. Head width 0.9 mm. Relative head measurements: width 50; length 64, clypeal length 17; lower interocular distance 32; upper interocular distance 33; interantennal distance 13; antennocular distance 5; interocellar distance 16; ocellocular distance 10. Anterior margin of clypeus truncate, upper margin slightly concave; other areas of clypeus and frons convex with depressions centrally, around the anterior ocelli and antennal implants; lower part of facial fovae broadened, upper part narrow, not bent towards ocelli; antennal scapes anteriorily flattened; malar space short; labial palp segments increasing in length in the order 2, 1, 3, 4. Clypeus and frons above antennae shiny with punctures wide apart; facial fovae, interocellar area and scutum with dense reticulation and dull.

Head black; antennae brown with flagella yellowish ventrally; labial palp segments 1,2 dark brown, segments 3, 4 light brown; legs yellowish with femora dark brown. First recurrent vein of forewing interstitial with first submarginal crossvein. Scattered long white hairs on frons, clypeus, antennal scapes, vertex, mandibles, posterior genae, sides of thorax, venter and gastral tergum 5.

*Male.* Length approximately 3.6 mm; wing length approximately 2.6 mm. Head width 0.8 mm. Relative head measurements: width 50; length 57, clypeal length 24; lower interocular distance 29; upper interocular distance 34; interantennal distance 10; antennocular distance 9; interocellar distance 14; ocellocular distance 10. Forewings and labial palps as in female; final three flagellar segments with indentations ventrally; inner hind tibial spur finely pectinate; frons above antennae shining with punctures wide apart. All labial segments dark; legs dark brown with fore tibia and all tarsae yellowish. Terminalia as in Fig. 5A–E.

**Figure 4.**
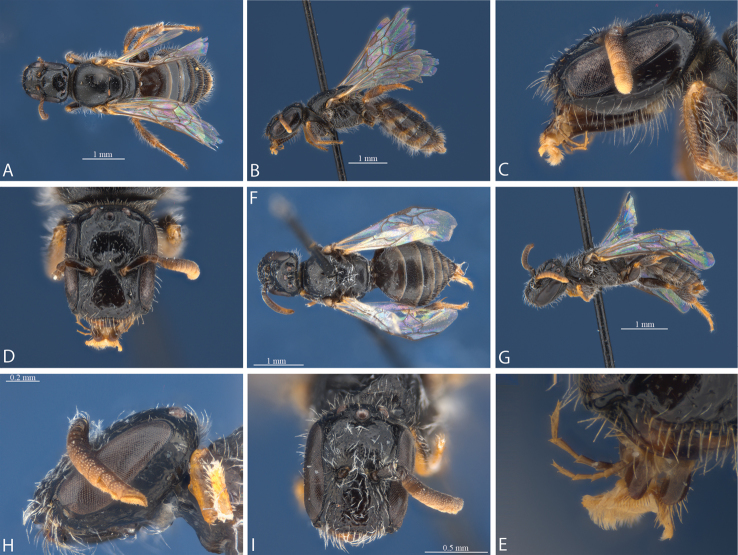
*Euhesma micans* sp. n.: **A–E** Holotype female: **A** dorsal **B** lateral **C** head lateral **D** head frontal **E** mouthparts **F–I** Allotype male: **F** dorsal **G** lateral **H** head lateral **I** head frontal.

**Figure 5.**
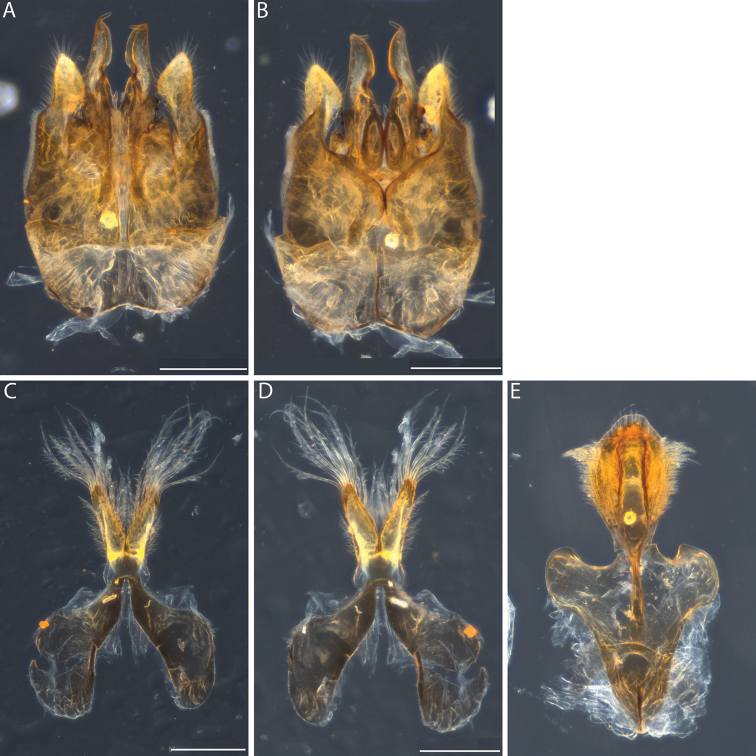
*Euhesma micans* sp. n.: **A–E** Allotype male genital structures: **A** genital dorsal **B** genital ventral **C** S7 dorsal **D** S7 ventral **E** S8 dorsal. Scale bars: 200 μm.

##### Etymology.

The specific name refers to the shiny frons.

#### Euhesma lyngouriae

Taxon classificationAnimaliaHymenopteraColletidae

Hogendoorn & Leijs sp. n.

http://zoobank.org/4809A089-9751-4AC1-B36D-7AC01F815ECA

Figs 6A–D

##### Material examined.

*Holotype:* ♀, SAMA 32-033284, Bon Bon Station, South Australia, 30.2389°S, 135.5098°E, 26 October 2010, R. Leijs, on flowers of *Swainsona stipularis* (SAMA). BOLD: AUSBS137-12.

♂, unknown.

##### Diagnosis.

Most like *Euhesma aurata*, however, forewing first recurrent vein interstitial with first submarginal crossvein, margin between clypeus and supraclypeal area lacking furrow, all abdominal segments amber, lacking brown bands.

##### Description.

*Female.* Length approximately 6.0 mm; wing length approximately 3.5 mm. Head width 1.6 mm. Relative head measurements: width 50; length 50; clypeal length 14; lower interocular distance 28; upper interocular distance 32; interantennal distance 12; antennocular distance 6; interocellar distance 12; ocellocular distance 8. Anterior margin of clypeus truncate, upper margin slightly concave, lacking median furrow; frons with median area elevated; facial fovae broad, upper part slightly bent towards ocelli; antennal scapes anteriorily flattened; malar space short; labial palp segments increasing in length in the order 1 = 3, 2. Clypeus and frons above antennae; facial fovae interocellar area and scutum with dense reticulation and dull. Head black; antennae brown with flagella yellowish ventrally; labial palp segments 1 dark brown, 2 and 3 yellowish; legs and gaster amber, tergal fovae and fore femora posteriorly dark brown. First recurrent vein of forewing interstitial with first submarginal crossvein. Scattered long white hairs on frons, clypeus, antennal scapes, vertex, mandibles, posterior genae, sides of thorax, venter and gastral tergum 5.

**Figure 6.**
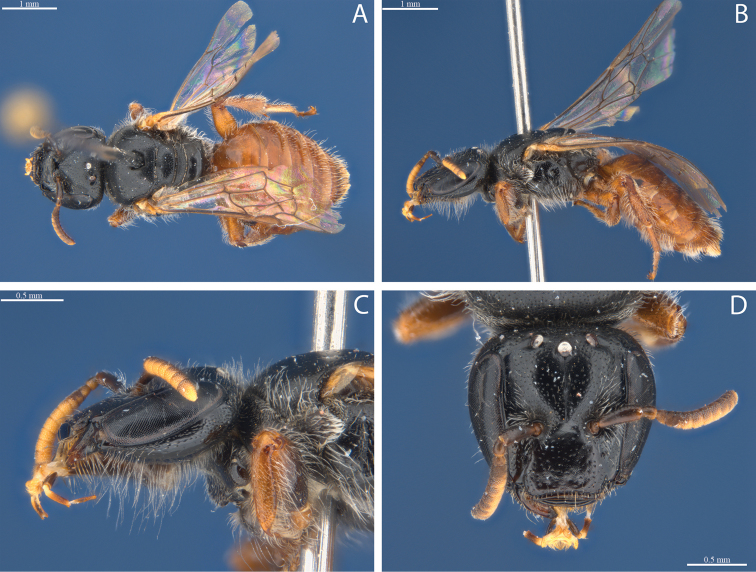
*Euhesma lyngouriae* sp. n.: **A–D** Holotype female: **A** dorsal **B** lateral **C** head lateral **D** head frontal.

##### Remarks.

The type lacks labial palp segment 4.

##### Etymology.

The specific name refers to the amber coloured legs and gaster of the female.

#### Euhesma aulaca

Taxon classificationAnimaliaHymenopteraColletidae

Hogendoorn & Leijs sp. n.

http://zoobank.org/A64B73ED-9553-4AB6-B0D1-B8095B482E0E

Figs 7A–I , 8A–E

##### Material examined.

*Holotype:* ♀, SAMA 32-033281, Bon Bon Station, South Australia, 30.8440°S, 135.5389°E, 27 October 2010, R Leijs (SAMA). BOLD: AUSBS133-12.

**Paratype** *(male).* ♂, SAMA 32-033282, same date and locality as holotype. *Paratypes:* 3♀, Bon Bon Station, South Australia, 30.7416°S, 135.3665°E, on *Eremophila scoparia*; 1♀, Bon Bon Station, South Australia, 30.5250°E, 135.5917°E; 7♀+1♂ in ethanol RL1636 all same date as holotype (SAMA). BOLD: AUSBS131,2,4-12.

##### Diagnosis.

The species is most like *Euhesma yellowdinensis*, but the clypeus has a distinctive deep median furrow and the total length of the labial palps is only slightly shorter than the head.

##### Description.

*Female.* Length approximately 5.5 mm; wing length approximately 3.5 mm. Head width 1.6 mm. Relative head measurements: width 50; length 50; clypeal length 14; lower interocular distance 28; upper interocular distance 32; interantennal distance 12; antennocular distance 6; interocellar distance 12; ocellocular distance 8. Clypeus with deep median furrow, anterior margin truncate, upper margin indistinct; facial fovae broad, upper part slightly bent towards ocelli; malar space short; labial palp segments increasing in length in the order 1,2,3=4. Clypeus and frons above antennae, interocellar area and scutum with dense reticulation; clypeus and supraclypeal area shining, frons, paraocular areas and scutum dull. Head black; antennae brown with flagella yellowish ventrally; labial palp segments 1 and 2 dark brown, 3 and 4 ribbon-like, yellowish; all femora and tibiae of middle and hind legs medially dark brown, fore tibiae, all tarsi and remaining parts yellow; gaster dark brown. Marginal zones of metasoma wide and translucent. Forewing with first recurrent vein almost interstitial with first submarginal crossvein. Scattered long white hairs on frons, clypeus, antennal scapes, vertex, mandibles, posterior genae, sides of thorax, venter and gastral tergum 5, pygidial fimbria pale orange.

*Male.* Length approximately 5.2 mm; wing length approximately 3.6 mm. Head width 1.2 mm. Relative head measurements: width 50; length 60, clypeal length 17; lower interocular distance 22; upper interocular distance 30; interantennal distance 8; antennocular distance 5; interocellar distance 12; ocellocular distance 9. Forewings, labial palps and other characters as in female; inner hind tibial spur roughly pectinate; Terminalia as in Fig. 8A–E.

**Figure 7.**
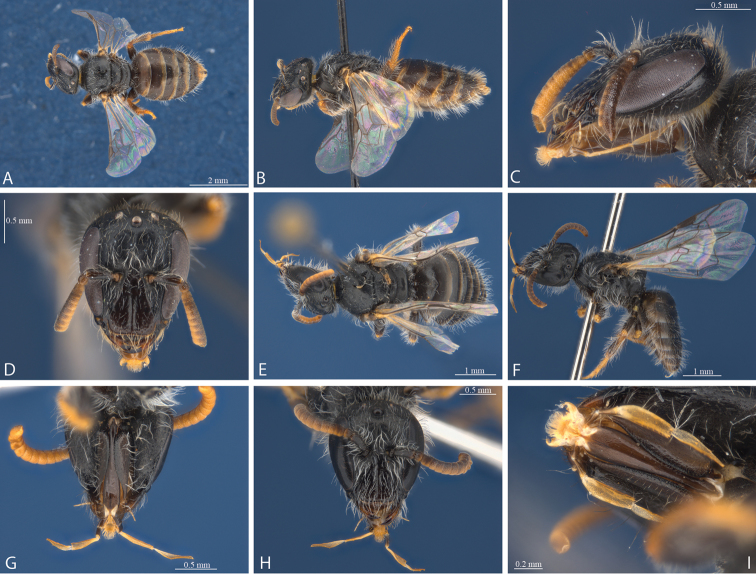
*Euhesma aulaca* sp. n.: **A–D, I** Holotype female: **A** dorsal **B** lateral **C** head lateral **D** head frontal **I** mouthparts **E–H**: Allotype male **E** dorsal **F** lateral **G** head ventral **H** head frontal.

**Figure 8.**
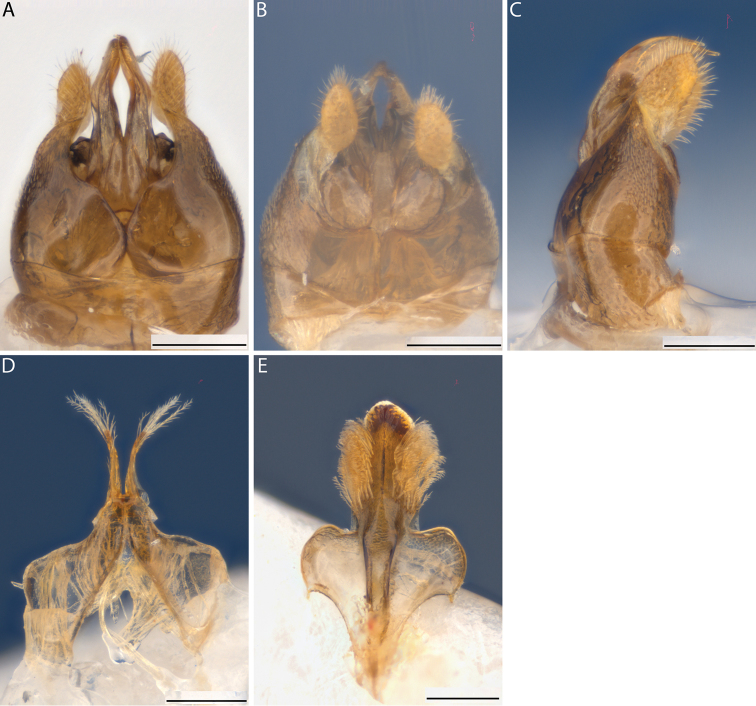
*Euhesma aulaca* sp. n.: **A–E** Allotype male genital structures: **A** genital dorsal **B** genital ventral **C** genital lateral **D** S7 dorsal **E** S8 dorsal. Scale bars: 200 μm.

##### Remarks.

Exley (1998) distinguishes the species from group 1 (the *alicia* species group) from those of groups 2 and 3 on the basis of the length of the labial palps, which are substantially shorter than the head in the species of group 1, and longer than the head in groups 2 and 3. Although the labial palps of female *Euhesma aulaca* are shorter than the head, the difference in length is only slight, while the shape and length of the labial palp segments are like other species in the *alicia* species group. Based on molecular and morphological data, *Euhesma aulaca* has been classified in the *alicia* species group, and the first couplet of the key has been modified to include this species.

##### Etymology.

The specific name means ‘with a furrow’, referring to the deep median furrow in the clypeus.

## Discussion

DNA barcoding has been used to associate the sexes of species. Furthermore, we used integrative taxonomy (Gibbs 2009; 2011) was used to identify species that were new to science. Four new species of *Euhesma* were found and described.

Despite the small size of the present molecular database, both in terms of numbers of species and numbers of nucleotides involved, it provides some insights with respect to validity of the *Euhesma* groups described by Exley (1998, 2001), of *Euhesma* as a genus, and of some other euryglossine clades.

While Exley (1998) noted that the *Euhesma* species groups associated with *Eremophila* would not necessarily be monophyletic, the molecular data so far support monophyly. The limited molecular data (Fig. 1) also support the *alicia* (group 1) and *atra* (group 2) species groups as recognized by Exley (1998). However, it should be noted that molecular data are only available for six of the 23 described species, and do not as yet include any representatives of the *coppinensis* species group.

By contrast, and in spite of the fact that the database is far from complete, the COI phylogeny presented here (Suppl. material 2, Fig. 1) suggests that *Euhesma* as a genus is paraphyletic. This is not surprising, as the genus was erected by Michener (1965) to include species that did not fit elsewhere, and Michener himself (2007) suggested that *Euhesma* could be paraphyletic. It is envisaged that with the addition of identified species and additional DNA sequences based on mitochondrial as well as nuclear genes, eventually a robust molecular and morphological revision of euryglossine genera currently grouped with *Euhesma* should be possible.

Support was also found for some other euryglossine clades. Although results of phylogenetic analyses based on just a small fragment of mitochondrial DNA (used here as the ‘barcoding region’) are often not informative above genus level, some well supported clades (posterior probabilities > 0.8) combine species within the genera *Euryglossula*, *Euryglossina* and *Pachyprosopis*, of which their sister clade appears to be the *Euhesma walkeriana* species group.

The creation of an open access molecular DNA barcoding project of Australian native bees enhances possibilities for scientists with molecular capabilities to document bee biodiversity (Schmidt et al. 2015) and will encourage and facilitate taxonomic work. The accession codes can also be included in other databases, such as Atlas of Living Australia and PaDil Australian Pollinators. The concatenation of molecular and morphological information for species discovery is becoming more accepted in recent years (e.g. Gibbs 2009; 2011; Schmidt et al. 2015), and will lead to better outcomes in our understanding of Australian native bees and their conservation.

## Supplementary Material

XML Treatment for Euhesma albamala

XML Treatment for Euhesma micans

XML Treatment for Euhesma lyngouriae

XML Treatment for Euhesma aulaca

## Supplementary materials

Supplementary material 1 Type localities of new species Data type: occurence Explanation note: localities where type specimens where caught. This dataset is made available under the Open Database License (http://opendatacommons.org/licenses/odbl/1.0/). The Open Database License (ODbL) is a license agreement intended to allow users to freely share, modify, and use this Dataset while maintaining this same freedom for others, provided that the original source and author(s) are credited.Katja Hogendoorn, Mark Stevens, Remko Leijs

Supplementary material 2 Phylogenetic relationships of euryglossine species based on CO1 sequence data Data type: gene tree Explanation note: tree based on CO1 sequence data. This dataset is made available under the Open Database License (http://opendatacommons.org/licenses/odbl/1.0/). The Open Database License (ODbL) is a license agreement intended to allow users to freely share, modify, and use this Dataset while maintaining this same freedom for others, provided that the original source and author(s) are credited.Katja Hogendoorn, Mark Stevens, Remko Leijs
