# Supplementary material for: DNA barcoding of euryglossine bees and the description of new species of Euhesma Michener (Hymenoptera, Colletidae, Euryglossinae)
Source: Zookeys. 2015 Sep 16;(520):41–59. doi: 10.3897/zookeys.520.6185 (PMC4591721; doi:10.3897/zookeys.520.6185)
Supplement: Supplementary material 2 — Phylogenetic relationships of euryglossine species based on CO1 sequence data [file zookeys-520-041-s002.pdf]

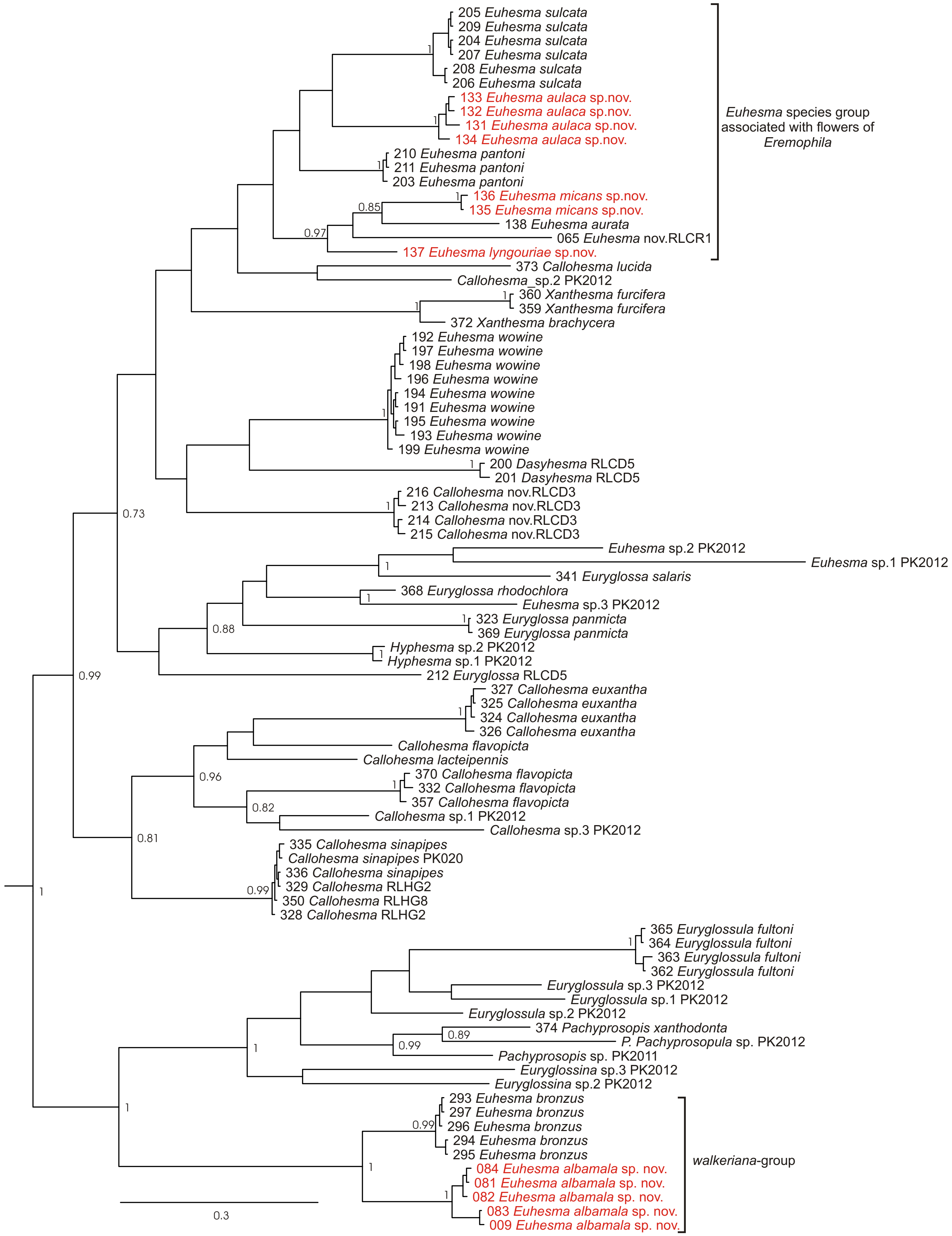

Figure S1.
Phylogenetic relationships of Euryglossine species based on BOLD and GenBank cytochrome oxidase subunit 1 sequence data, analysed using MrBayes (GTR-inv+gamma, partitioned by codon, 8M generations). Posterior probabilities for nodes are shown when > 0.7. The three digits preceding taxon names refer to BOLD:AUSBS###-12/13 specimens, other specimens are from GeneBank.
